# An empirical model for educational simulation of cervical dilation in first-stage labor

**DOI:** 10.1186/s41077-018-0068-3

**Published:** 2018-06-15

**Authors:** Silvano R. Gefferie, Anouk W. J. Scholten, Kim A. E. Wijlens, M. Luísa Ferreira Bastos, M. Beatrijs van der Hout-van der Jagt, Hans Zwart, Willem J. van Meurs

**Affiliations:** 1 Technical Medicine, University of Twente, Drienerlolaan 5, 7522 NB Enschede, The Netherlands; 2 Animals in Science, Eurogroup for Animals, Hertogstraat 29, 1000 Brussels, Belgium; 3 Department of Signal Processing Systems, Faculty of Electrical Engineering, Eindhoven University of Technology, PO Box 513, 5600 MB Eindhoven, The Netherlands; 4 Department of Applied Mathematics, Faculty of Electrical Engineering, Mathematics, and Computer Science, University of Twente, Drienerlolaan 5, 7522 NB Enschede, The Netherlands; 5 Cardiovascular and Respiratory Physiology Group, Faculty of Science and Technology, University of Twente, Drienerlolaan 5, 7522 NB Enschede, The Netherlands

**Keywords:** Model, First stage labor, Oxytocin, Uterine contractions, Cervical dilation, Educational simulation

## Abstract

**Background:**

Several models for educational simulation of labor and delivery were published in the literature and incorporated into a commercially available training simulator (CAE Healthcare Lucina). However, the engine of this simulator does not include a model for the clinically relevant indicators: uterine contraction amplitude and frequency, and cervical dilation. In this paper, such a model is presented for the primigravida in normal labor.

**Methods:**

The conceptual and mathematical models represent oxytocin release by the hypothalamus, oxytocin pharmacokinetics, and oxytocin effect on uterine contractions, cervical dilation, and (positive) feedback from cervical dilation to oxytocin release by the hypothalamus.

**Results:**

Simulation results for cervical dilation are presented, together with target data for a normal primigravida. Corresponding oxytocin concentrations and amplitude and frequency of uterine contractions are also presented.

**Conclusion:**

An original empirical model for educational simulation of oxytocin concentration, uterine contractions, and cervical dilation in first-stage labor is presented. Simulation results for cervical dilation match target data for a normal patient. The model forms a basis for taking into account more independent variables and patient profiles and can thereby considerably expand the range of training scenarios that can be simulated.

## Introduction

Uterine contractions, fetal descent, and cervical dilation are used to assess progression of labor. These related phenomena vary greatly among parturients and may have an incidence on the condition of the fetus. Simulators allow for practice of normal and critical situations at will and at no risk to real patients. Several models for educational simulation of labor and delivery were published in the literature [1–3] and incorporated into a commercially available training simulator [4]. The cited models make amplitude, frequency, duration, and resting tone of the uterine pressure waveform evolve spontaneously and under the influence of oxytocin and tocolytics. However, the relationships between these variables and cervical dilation are scripted.

A positive feedback loop involving oxytocin plays a central role in the progression of first-stage labor. The hypothalamus releases oxytocin into the bloodstream, which is transported to the uterus and stimulates uterine contractions. The contractions result in descent of the fetus. The descending fetal head causes cervical dilation. Cervical stretch receptors send a signal to the hypothalamus via afferent nerves, stimulating additional release of oxytocin, thereby closing the loop. This loop results in progressively intensifying contractions and dilation over time [5].

A database for a number of contraction and dilation profiles could be established and used for scripted training simulation purposes. However, realistic simulation of the interaction between oxytocin concentrations, uterine contractions, fetal descent, and dilation, for a number of patient profiles, and possibly under external influences such as administration of exogenous oxytocin and tocolytics, requires a model-based approach. Such a model would not only increase insight in the interacting processes, but also greatly expand the range of scenarios that can be simulated beyond the relatively simple phenomena that can be characterized in a database.

In this innovation paper, we lay the foundation for such a model by proposing an empirical model that simulates oxytocin concentrations and contraction and dilation profiles. The model should be able to run in real, or accelerated, time and should be easy to manipulate to reflect different patients.

## Methods

**Fig. 1:**
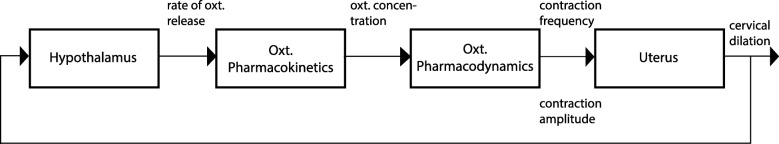
Conceptual model for educational simulation of cervical dilation during first-stage labor. Oxt.: oxytocin

Basic assumptions are made, similar to the ones in the article of Bastos et al. [3] and based on understanding of physiological relations, in order to keep the model as simple as possible, while approaching the target data of Zhang et al. [6]. The subsystems and variables connecting them, as described in the introduction, are reflected in the block diagram of Fig. 1.

The model equations referring to Fig. 1 from left to right are introduced below. The rate of oxytocin release by the hypothalamus *r(t)* in mU/min has a fixed component, reflected in the parameter *P*_*1*_ and depends linearly via a gain *P*_*2*_ on the sensed dilation *d(t)* in centimeters:

1 $$ r(t)={P}_1+{P}_2d(t) $$

Units of parameters can easily be derived from the units of the variables. The change in mass of oxytocin depends on the elimination rate constant *P*_*3*_, the current mass *m(t)* in mU, and the release rate, represented by a first-order pharmacokinetic model:

2 $$ \dot{m}(t)=-{P}_3\mathrm{m}(t)+r(t) $$

Dividing the mass by the volume of distribution *P*_*4*_ results in the concentration of oxytocin in mU/mL:

3 $$ c(t)=\frac{m(t)}{P_4} $$

The concentration dependency of contraction frequency *f(t)* in 1/min and contraction amplitude *a(t)* in mm Hg is represented by a sigmoidal curve, Eqs. (4) and (5). For example in Eq. (4), for a concentration equal to zero, the frequency is zero; for a concentration equal to *P*_*6*_, it is 50% of the maximum frequency *P*_*5*_; and for higher concentrations, it tends toward *P*_*5*_. The slope of both pharmacodynamic relationships is governed by *P*_*7*_:

4 $$ f(t)={P}_5\frac{c{(t)}^{P_7}}{{P_6}^{P_7}+\mathrm{c}{(t)}^{P_7}\ } $$

The amplitude has a non-zero baseline *P*_*8*_ and a concentration-dependent part reflected in *P*_*9*_ (the difference between the maximum and baseline amplitude values):

5 $$ a(t)={P}_8+{P}_9\frac{\mathrm{c}{(t)}^{P_7}}{{P_6}^{P_7}+c{(t)}^{P_7}\ } $$

The change in dilation depends on the pressure exerted by the fetus on the cervix *P*_*10*_, which is assumed fixed in this version of the model, and on the product of frequency and amplitude. This second influence is assumed linearly proportional with gain *P*_*11*_. Thus, the resulting uterine model is

6 $$ \dot{d}(t)={P}_{10}+{P}_{11}\mathrm{f}(t)\mathrm{a}(t) $$

Referenced and empirically adjusted parameters are listed in Table 1.

**Table 1:** Model parameters. For consistency, all values are given in three significant digits

Symbol	Description	Value	Unit	Reference
P_1_	Baseline oxytocin release rate in hypothalamus	0.740	mU/min	[7]
P_2_	Gain from cervical dilation to oxytocin release rate in hypothalamus	50.0	mU/(min cm)	
P_3_	Pharmacokinetic parameter: Elimination rate constant	0.0693	1/min	[3]
P_4_	Pharmacokinetic parameter: Volume of distribution	18,700	mL	[8, 9]
P_5_	Maximal concentration frequency	0.500	1/min	[2]
P_6_	Pharmacodynamic parameter: Oxytocin concentration resulting in 50% effect	7.90	mU/mL	[3]
P_7_	Pharmacodynamic parameter: Slope of sigmoidal curve	1.11	dimensionless	[2]
P_8_	Baseline contraction amplitude	40.0	mm Hg	[10]
P_9_	Maximal contraction amplitude	40.0	mm Hg	[2]
P_10_	Dilation increase due to pressure exerted by the fetus on the cervix	1.00 × 10^−3^	cm/min	[11]
P_11_	Dilation increase due to contraction frequency and amplitude	1.90 × 10^−2^	cm/mm Hg	

Equations (1–6) were implemented in MATLAB (MATLAB R2017a, The MathWorks Inc.) using the Euler forward method with an integration step size of 1 min to integrate the two differential equations. Model simulation results are presented and compared to target data obtained from Zhang et al. [6] for cervical dilation.

## Results

**Fig. 2:**
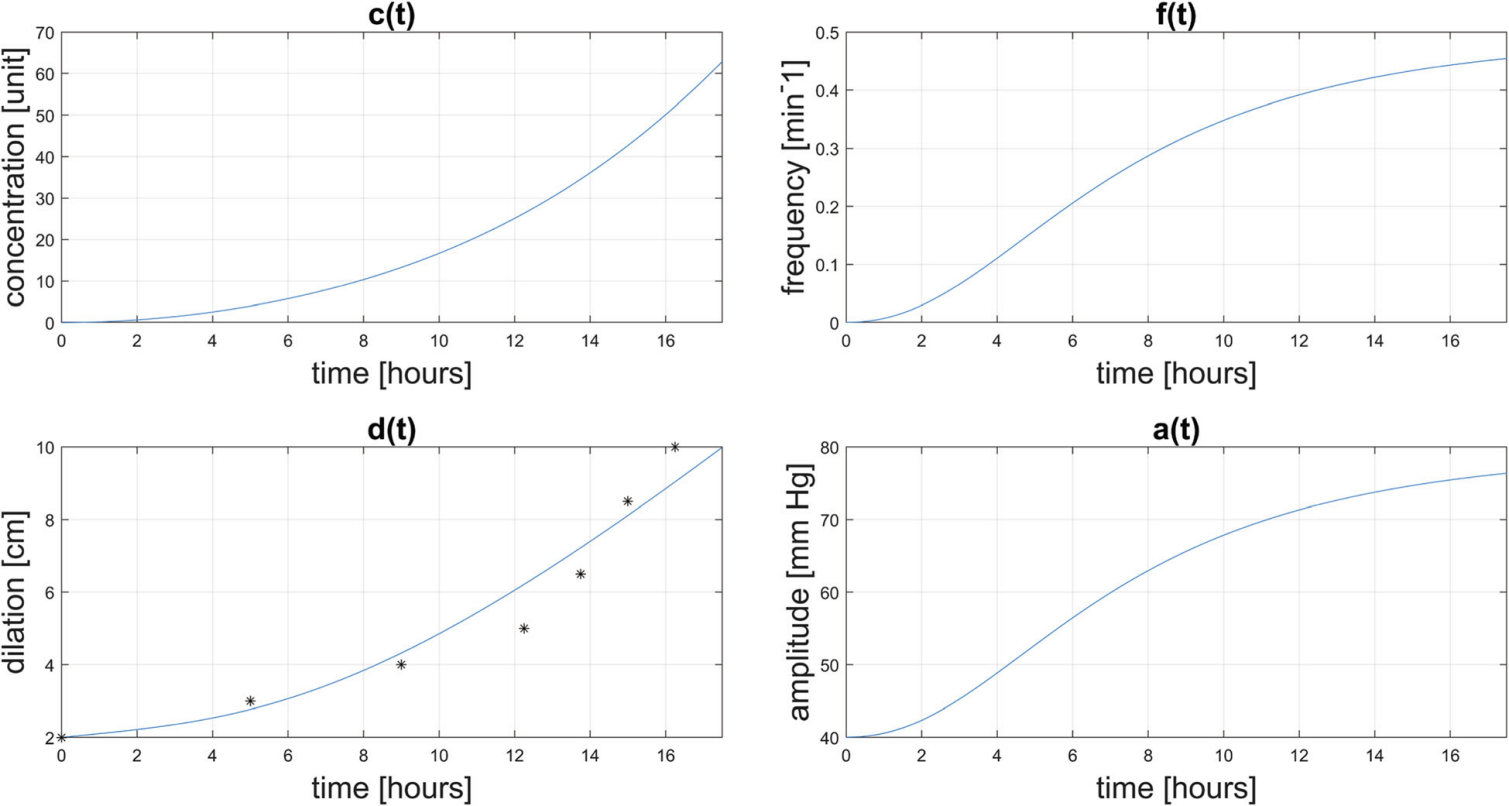
Simulation results and dilation target data

Figure 2 shows simulation results from the initial conditions *d(0)* = 2.00 cm, and *m(0)* = 275 mU, using the parameters listed in Table 1. The stars in Fig. 1 correspond to dilation data for patient A in Zhang et al. [6]. This patient was admitted at 2 cm and labor progressed to 10 cm with labor considered non-protracted.

## Discussion

Simulated dilation matches dilation in a normal patient in an approximation that is considered realistic enough for educational simulation. Parameters *P*_*2*_ and *P*_*11*_ were adjusted to achieve this match. Further research could address which parameters are patient- or population-specific and can be used to simulate different labor types. The model parameter estimation procedure could then be formalized so that a clinical instructor can easily use it to meet his or her educational objectives. Ongoing work involves expanding the set of target data and matching simulation results to them, to enhance model validity and demonstrate the possibility to match different patient profiles. Critical analysis of the pharmacokinetic model may be necessary. It would also be interesting to explore validity of the model for exogenous oxytocin administration.

## Conclusion

An original empirical model for educational simulation of oxytocin concentrations, uterine contractions, and cervical dilation in first-stage labor is presented. Simulation results for cervical dilation match target data for a normal patient. The proposed model forms a sound, explicit basis for taking into account more independent variables and patient profiles, and thereby considerably expand the range of training scenarios that can be simulated.
