# Correction to: An empirical model for educational simulation of cervical dilation in first stage labor

**DOI:** 10.1186/s41077-021-00184-y

**Published:** 2021-10-01

**Authors:** Silvano R. Gefferie, Anouk W. J. Scholten, Kim A. E. Wijlens, M. Luísa Ferreira Bastos, M. Beatrijs van der Hout-van der Jagt, Hans Zwart, Willem L. van Meurs

**Affiliations:** 1 Technical Medicine, University of Twente, Drienerlolaan 5, 7522 NB Enschede, The Netherlands; 2 Animals in Science, Eurogroup for Animals, Hertogstraat 29, 1000 Brussels, Belgium; 3 Department of Signal Processing Systems, Faculty of Electrical Engineering, Eindhoven University of Technology, PO Box 513, 5600 MB Eindhoven, The Netherlands; 4 Department of Applied Mathematics, Faculty of Electrical Engineering, Mathematics, and Computer Science, University of Twente, Drienerlolaan 5, 7522 NB Enschede, The Netherlands; 5 Cardiovascular and Respiratory Physiology Group, Faculty of Science and Technology, University of Twente, Drienerlolaan 5, 7522 NB Enschede, The Netherlands

**Correction to: Adv Simul 3:9 (2018)**

**https://doi.org/10.1186/s41077-018-0068-3**

Continuing work on a recently published empirical model for educational simulation of cervical dilation [1] resulted in identification of errors in the code implementing this model. Numerical values of three parameters and one state variable had to be updated to obtain the original simulation results with corrected code. The errors identified in the original code included incorrect assignment of the value of the parameter that governs the dilation increase due to pressure exerted by the fetus on the cervix, a discrete time step specified in hours with parameters using minutes as a time reference, numerical integration of a static equation, and unnecessary capping of the uterine contraction amplitude. In the MATLAB code listed in the appendix, these errors are corrected. To obtain the originally published simulation results for cervical dilation, three parameter values had to be adjusted, see Table 1.

**Table 1:** Original and updated model parameters. See [1] for a detailed description of the individual parameters and references to numerical values

	original value	updated value	units
P_1_	0.740		mU/min
P_2_	50.0	1.70	mU/(min cm)
P_3_	0.0693		1/min
P_4_	18,700		mL
P_5_	0.500		1/min
P_6_	7.90	0.00610	mU/mL
P_7_	1.11		dimensionless
P_8_	40.0		mm Hg
P_9_	40.0		mm Hg
P_10_	1.00 × 10^−3^		cm/min
P_11_	1.90 × 10^−2^	4.00 × 10^−4^	cm/mm Hg
m(0)	273	59.0	mU
d(0)	2.0		cm

On closer inspection, it was also found that the value of the parameter AFR_50_ of 7.9 mU/min in [2] was incorrectly assigned to P_6_, which is a concentration in mU/mL. In semi-steady state it can be derived from Eqs. (2, 3) of the original paper that the updated value listed in Table 1 corresponds to the concentration in steady state on an infusion of magnitude AFR_50_ for the given pharmacokinetic parameters P_3_ and P_4_. The evolution of drug mass over time is given by the pharmacokinetic equation, Eq. (4) of the original paper. In semi-steady state, drug mass is proportional to infusion rate. This value is assigned to m (0) in Table 1. Simulation results for cervical dilation using corrected code and adjusted numerical values match the results presented in the original paper in good approximation. The conceptual model and all presented model equations stood up to this additional scrutiny.
